# A nomogram to predict overall survival and disease-free survival after curative-intent gastrectomy for gastric cancer

**DOI:** 10.1007/s13304-021-01083-7

**Published:** 2021-06-14

**Authors:** Alice Sabrina Tonello, Giulia Capelli, Quoc Riccardo Bao, Alberto Marchet, Fabio Farinati, Timothy M. Pawlik, Dario Gregori, Salvatore Pucciarelli, Gaya Spolverato

**Affiliations:** 1 First Surgical Clinic, Department of Surgical, Oncological and Gastroenterological Sciences (DiSCOG), University of Padua, Padua, Italy; 2 Gastroenterology Unit, Department of Surgical, Oncological and Gastroenterological Sciences (DiSCOG), University of Padua, Padua, Italy; 3 Department of Surgical Oncology, The Ohio State University Wexner Medical Center, Columbus, OH USA; 4 Unit of Biostatistics, Epidemiology, and Public Health, Department of Cardiac, Thoracic, and Vascular Sciences, University of Padua, Padua, Italy

**Keywords:** Gastric cancer, Nomogram, Overall survival, Disease-free survival

## Abstract

An individual prediction of DFS and OS may be useful after surgery for gastric cancer to inform patients and to guide the clinical management. Patients who underwent curative-intent resection for gastric cancer between January 2010 and May 2020 at a single Italian institution were identified. Variables associated with OS and DFS were recorded and analysed according to univariable and multivariable Cox models. Nomograms predicting OS and DFS were built according to variables resulting from multivariable Cox models. Discrimination ability was calculated using the Harrell’s Concordance Index. Overall, 168 patients underwent curative-intent resection. Nomograms to predict OS were developed including age, tumor size, tumor location, T stage, N stage, M stage and post-operative complications, while nomogram to predict DFS includes Lauren classification, and lymph node ratio (LNR). On internal validation, both nomograms demonstrated a good discrimination with a Harrell’s C-index of 0.77 for OS and 0.71 for DFS. The proposed nomogram to predict DFS and OS after curative-intent surgery for gastric cancer showed a good discrimination on internal validation, and may be useful to guide clinician decision-making, as well help identify patients with high-risk of recurrence or with a poor estimated survival.

## Introduction

Gastric cancer (GC) is the fifth most common tumor and the third leading cause of cancer-related mortality worldwide, accounting for over 1,000,000 new cases and 783,000 deaths worldwide in 2020 [1]. In Italy, 14,500 new cases of GC and 8700 GC-related deaths have been estimated to be in 2020 [2].

Gastrectomy with adequate lymphadenectomy represents the gold standard for treatment of resectable disease. Although surgery offers the best chances of curative treatment for GC, recurrences occur in 20–50% of patients after surgery [3]. In fact, recurrence typically occurs within 3 years of surgery and is associated with a poor prognosis [3–5]. Traditionally, depth of tumor invasion, nodal metastasis, lymphovascular invasion and Lauren’s classification are the main risk factors associated with recurrence [3–6].

Accurate staging systems are crucial to assess prognosis and recommend chemotherapy or close surveillance. However, the TNM staging system has been criticized for grouping patients within the same stage despite these often have different prognosis [7, 8]. The N stage, which stratifies patients according to the number of metastatic lymph nodes retrieved at surgery, was reported to be an important prognostic factor affecting survival. For this reason, a patient undergoing an inadequate nodal dissection may be under-staged, and subsequent survival predictions would be inaccurate [9, 10]. Beyond TNM stage, GC survival is affected by several other factors, such as demographic factors (e.g. age, gender, genetic predisposition), comorbidities, tumor characteristics (e.g. size, location, Lauren type), as well as the role of chemotherapy and post-operative complications [11, 12]. Therefore, a prognostic system reporting purely data on tumor depth, nodal status and the presence of metastasis will be incomplete and deficient of several elements affecting prognosis [13, 14].

More recently, efforts have increasingly focused on developing improved prognostic systems to offer a more accurate prediction of long-term prognosis and risk of recurrence [15, 16]. In particular, nomograms have been increasingly adopted within the oncological field for a variety of tumors. Nomograms are graphical representations of complicated algorithms that are able to estimate survival of an individual patient by combining information on demographics and tumor characteristics with data on depth of tumor invasion, nodal status and metastasis provided by the TNM staging system. Nomograms can be helpful both in the pre-operative setting to estimate the risk of lymph node metastasis, as well as in the post-operative setting to estimate overall survival (OS) and recurrence risk [8]. While several nomograms are available for GC, most of these nomograms were based exclusively on data from United States or Asia [7, 11, 16–18]. To do, GC nomogram based on European data is lacking, and we sought to develop a novel nomogram based on a 10-year single-institution experience in GC management.

The aim of the current study was to give an individual prognostication of recurrence risk and survival using these graphical algorithms.

## Methods

### Data collection

All the patients who underwent curative-intent resection for GC from January 2010 to May 2020 were retrospectively collected.

Inclusion criteria were primary histologically proven gastric cancer and curative-intent surgery (R0-R1). Gastroesophageal Siewert type III tumors were included in the study, while types I and II were excluded. Patients who underwent surgery with a palliative intent (R2) or urgent/emergent surgery were also excluded. Data on demographics (i.e. age, gender, BMI, comorbidity, familiar history of gastric cancer, *Helicobacter pylori* infection), symptoms (bleeding, obstructive symptoms) and pre-operative work-up (endoscopy, CT scan, endoscopic ultrasound) were collected.

GC resection was performed using an open approach and grouped according to the type of resection (total, distal, proximal gastrectomy, extended total gastrectomy, esophagogastric resection, pancreaticoduodenectomy, remnant gastrectomy). Remnant gastrectomy was performed in patients with a previous gastric resection for benign disease or gastric remnant novel tumors. Surgical procedures other than gastric resection, such as esophagogastric resection, pancreaticoduodenectomy or multivisceral resection, were performed to achieve negative resection margins at final pathology. Data regarding chemotherapy regimens and radiation therapy, both perioperative and adjuvant, were also recorded. Post-operative complications occurring within 30 days from surgery were classified according to Clavien–Dindo classification, and categorized into minor complications (i.e. Clavien–Dindo 0–2) and major complications, (i.e. Clavien–Dindo 3–5) [19].

Lymphadenectomy was classified as D1 or D2 according to Japanese Gastric Cancer Association (JGCA 5th ed.) guidelines [20]. A D2 lymphadenectomy was performed when a locally advanced disease or nodal metastasis were clinically and/or intra-operatively suspected. Lymph node status was recorded considering the presence or absence of metastatic nodes at any nodal station (from n.1 to n.12.), the total number of nodes retrieved in the specimen, the total number of metastatic nodes, and the lymph nodes ratio (LNR).

Data concerning histopathological examination were recorded including margin status (R0: no cancer at resection margins, R1: microscopical residual cancer), tumor histological subtype according to the WHO classification, tumor size, lymphovascular invasion, Lauren’s classification, Ming classification and grading. The 8th edition of AJCC/UICC TNM was used for cancer staging [21]. Data prior to 2017 were updated to the 8th edition of TNM staging system.

Follow-up was obtained matching clinical visits recorded in electronic archives of Surgical and Oncological divisions. Follow-up time was calculated from the date of surgery to the date of last contact. Disease-free survival (DFS) was defined as the time from surgery to the first documented recurrence. Overall survival (OS) was defined as the time from operation to death or last follow-up. The pattern of recurrence was categorized as local (i.e. recurrence involving anastomosis or gastric remnant), nodal (including both loco-regional and distant nodes) or distant (i.e. peritoneum, liver, lung, bone or multiple sites).

### Statistical analysis

Descriptive statistics were reported as absolute number percentages for categorical variables, while continuous variables were expressed as median values with interquartile ranges (IQR). OS and DFS were calculated from the date of surgery to the date of the event (local or distant recurrence, death, or the last follow-up), and were evaluated using Kaplan–Meier method. Variables associated with OS and DFS were recorded and analysed according to univariable and multivariable Cox models. Results were reported as Hazard Ratio (HR), 95% CI and *p* value. A *p* value < 0.05 was considered statistically significant.

The primary end-point of the study was to create nomograms to predict OS and DFS. Nomograms predicting OS and DFS were built according to variables resulting from multivariable Cox models. The performance of the two models was internally cross-validated via bootstrap resampling procedure with 10,000 replicates to quantify any overfitting. Discrimination ability was calculated using the Harrell’s Concordance Index [22], which is a proxy of the concordance between predicted and observed outcomes. C-index values within 0.7–0.8 indicated a good discrimination, while values > 0.8 indicated an excellent discrimination.

All the analyses were performed using R software (version 4.0.3) [23] with the packages survival and rms [24].

## Results

### Patients’ demographic and clinical characteristics

Overall, 168 patients were collected (Table 1). Median patient age was 71 years (IQR 62–77), and 57.1% (*n* = 96) of patients were male. Median BMI was 24.1 kg/m2 (IQR 22.4–28.4). Familial history of gastric cancer was present in 9.6% (*n* = 16) of patients. On EGD, most tumors were located in the antrum (*n* = 87; 51.8%) or gastric body (*n* = 41; 24.4%), while fewer were located in the fundus (*n* = 14; 8.3%) or at the gastroesophageal junction (Siewert III) (*n* = 16; 9.5%). A minority of patients received perioperative chemotherapy (*n* = 19; 11.0%), while 93 patients (55.4%) received adjuvant chemotherapy following resection.

**Table 1:** Patients’ demographic, clinical, and treatment characteristics

Variables	*N* = 168 (% or IQR)
Age years, median (IQR)	71 (62–77)
Gender	
Female	72 (42.9)
Male	96 (57.1)
Preoperative CEA (> 4ug/L)	54 (32.1)
BMI kg/m2, median (IQR)	24.1 (22.4–28.4)
Tumor size mm, median (IQR)	40 (30–60)
Tumor location	
Cardia (Siewert III type)	16 (9.5)
Fundus	14 (8.3)
Body	41 (24.4)
Antrum	87 (51.8)
Pylorus	1 (0.6)
Multicentric disease	2 (1.2)
Gastric remnant	7 (4.2)
Type of surgical resection	
Subtotal gastrectomy	76 (45.2)
Total gastrectomy	66 (39.3)
Extended total gastrectomy	12 (7.1)
Remnant gastrectomy	7 (4.2)
Proximal gastrectomy	1 (0.6)
Esophageal resection	5 (3.0)
Multivisceral resections	17 (10.1)
Extent of lymphadenectomy	
D1	44 (26.2)
D2	124 (73.8)
Adjuvant treatment	
Chemotherapy	93 (55.4)
Radiotherapy	13 (7.7)
Length of stay, days, median (IQR)	11 (10–13)
Post-operative complications	69 (41)
Clavien–Dindo classification	
Grade 0–2	150 (89.3)
Grade 3–5	18 (10.7)
Deep abdominal collections	23 (13.7)
Bleeding requiring transfusions	18 (10.7)
Anastomotic leakage	8 (4.8)

*IQR* interquartile range, *CEA* carcinoembryonic antigen, *NOS* not otherwise specified, *SRG* signet ring cell, *TNLE* total number of nodes examined, *LNR* lymph node ratio

Distal gastrectomy was performed in 76 patients (45.2%), total gastrectomy in 66 (39.3%). The remaining patients underwent an extended total gastrectomy (*n* = 12; 7.1%), a proximal gastrectomy (*n* = 1; 0.6%) and a resection of the gastric remnant (*n* = 7; 4.2%), pancreaticoduodenectomy (*n* = 1; 0.6%), esophagogastric resection (*n* = 5; 3.0%). Multivisceral resections were performed in 17 (10.1%) cases. Splenectomy was the most common procedure, occurring in 12 (7.1%) patients, followed by distal pancreatectomy in 6 (3.6%) patients, and colon/bowel resection in 6 (3.6%) patients. D1 lymphadenectomy was performed in 44 (26.2%) patients, while D2 lymphadenectomy in 124 (73.8%) patients.

Among patients who underwent remnant gastrectomy, 5 patients had a previous surgery for benign disease (i.e. gastric and duodenal ulcers), while 2 patients had a history of tumor. Specifically, one patient underwent distal gastrectomy for GIST in 1999 with a negative follow-up until 2015, the other had a distal gastrectomy for a T2N2 GC followed by 6 cycles of adjuvant chemotherapy in 2009, with a negative follow-up until December 2018. Considering the long time between these two malignancies, the second neoplasm was considered a novel tumor instead of a local recurrence.

Median length of hospital stay was 11 days (IQR 10–13). Post-operative morbidity occurred in 41% of patients (*n* = 69). Pulmonary complications (*n* = 33; 19.6%) were the most frequent, followed by deep intraabdominal collections (*n* = 23; 13.7%) and post-operative bleeding requiring blood transfusions (*n* = 18; 10.7%). Anastomotic leakage occurred in 4.8% of patients (*n* = 8). Cardiovascular, thromboembolic complications, and duodenal stump leak and bowel perforation occurred in less than 5% of patients. A minority of patients (*n* = 12; 7.1%) needed a radiological procedure (i.e. Clavien–Dindo grade 3a) or re-intervention (i.e. Clavien–Dindo 3b) to treat post-operative complications. A total of 5 (3.0%) patients developed single or multi-organ dysfunction (i.e. Clavien–Dindo grade 4a and 4b). Post-operative mortality rate was 0.6% (*n* = 1).

### Pathological analysis and long-term outcomes

On histopathological examination (Table 2), 72 (48.6%) patients had a diffuse type tumor, while the remaining were either intestinal (*n* = 67; 45.3%) or mixed (*n* = 9; 6.1%) type. Median tumor size was 40 mm (IQR 30–60). The majority of patients had poorly differentiated or undifferentiated tumors (*n* = 94; 65.3%), and most were classified as tubular (*n* = 68; 40.5%) or poorly cohesive (including signet ring cell, *n* = 56; 33.3%) subtypes. Pathological locally advanced stage was found in 49 (29.2%), 51 (30.4%), and 11 (6.5%) in pT3, pT4a and pT4b, respectively. Overall, most patients had stage III disease (*n* = 79; 47.0%); whereas 43 (25.6%) and 45 (26.8%) of patients had stage I and II disease, respectively.

**Table 2:** Histopathological characteristics

Variables	*N* = 168 (% or IQR)
Histotype	
Tubular	68 (40.5)
Poorly cohesive (NOS or SRC)	56 (33.3)
Other types	44 (26.2)
Lauren’s classification	
Mixed	9 (6.1)
Intestinal	67 (45.2)
Diffuse	72 (48.6)
Histologic grade (*n* = 144)	
G1-G2	50 (29.8)
G3	94 (56.0)
NA	24 (14.3)
T stage	
T1	37 (22.0)
T2	20 (11.9)
T3	49 (29.2)
T4	62 (36.9)
N stage	
N0	67 (39.9)
N1	28 (16.7)
N2	19 (11.3)
N3a	25 (14.9)
N3b	29 (17.3)
M stage	
M1	1 (0.6)
TNM Stage	
Stage I	43 (25.6)
Stage II	45 (26.8)
Stage III	79 (47.0)
Stage IV	1 (0.6)
Total number of nodes examined median (IQR)	31.5 (21–43)
Lymph node ratio (LNR) median (IQR)	0.06 (0–0.28)
Lymphatic invasion	
Present	109 (65.9)
NA	11 (6.5)
Vascular invasion	
Present	67 (41.6)
NA	7 (4.2)
Radicality	
R0	158 (94.0)
R1	10 (5.9)

*IQR* interquartile range, *NOS* not otherwise specified, *SRG* signet ring cell, *LNR* lymph node ratio

Microscopically, tumor infiltration (i.e. R1) was found in 10 (5.9%) patients, since they had a positive resection margins (gastric, esophageal or duodenal). Vascular and lymphatic invasion was reported in 41.6% (*n* = 67) and 69.4% (*n* = 109) patients, respectively. The median of the total number of nodes examined was 31.5 (IQR 21–43). Lymph node metastasis was found in 101 (60.1%) patients, resulting in an N1 stage in 28 (16.7%) patients, N2 stage in 19 (11.3%), N3a in 25 (14.9%), and N3b in 29 (17.3%). Median LNR was 0.06 (IQR 0–0.280).

The median follow-up for our cohort was 20.1 months (IQR 8.2–49.5). During follow-up, 60 (35.7%) patients experienced a recurrence, and 73 (43.5%) patients died. Overall, 3 (1.7%) patients had local recurrence, 10 (5.9%) patients had a nodal recurrence, and 47 (28.0%) had distant recurrence. The sites of distant recurrence were peritoneum in 30 patients, liver in 8, lung in 1, and multiple in 8.

**Fig. 1:**
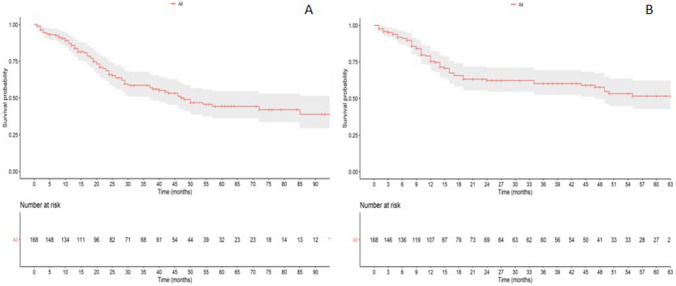
Kaplan–Meier curves demonstrating overall survival **a** and disease-free survival **b** for patients following resection for primary gastric cancer

The 1-, 3- and 5-year DFS was 75.3% (95% CI 68.6–82.7), 60.2% (95% CI 52.4–69.3) and 51.6% (95% CI 42.8–62.3), respectively. The 1-, 3- and 5-year OS was 85.7% (95% CI 80.4–91.5), 58.7% (95% CI 50.9–67.7) and 44.2% (95% CI 36.0–54.5), respectively (Fig. 1).

### Model specification and predictors of overall and disease-free survival

Cox regression analysis results are reported in Tables 3 and 4. At univariable analysis, pre-operative CEA, preoperative clinical locally advance disease, margin status (R1), number of total lymph nodes dissected, lymph nodes metastases, LNR, Lauren’s classification, vascular and lymphatic invasion, T and N stage, TNM stage, thromboembolic complications, and neoadjuvant and adjuvant chemotherapy were significantly associated with DFS. At multivariable analysis, Lauren’s mixed type compared with the diffuse type (HR 3.01, 95% CI 1.22–7.42, *p* = 0.017), and LNR (HR 2.13, 95% CI 1.63–2.79, *p* < 0.001) were independently predictor of DFS.

**Table 3:** Variables associated with Disease-Free Survival (DFS) according to the Cox proportional hazards regression model

Variables	Univariate analysis			Multivariate analysis		
	HR	95% CI	*p *value	HR	95% CI	*p* value
Age	0.92	0.68–1.24	0.590	0.95	0.67–1.34	0.788
LNR	2.01	1.60–2.53	< 0.001	2.13	1.63–2.79	0.029
Lauren’s classification Intestinal vs diffuse	0.53	0.29–0.96	0.036	0.87	0.46–1.65	0.691
Mixed vs diffuse	1.99	0.83–4.77	0.122	3.01	1.22–7.42	0.017
Preoperative CEA > 4ug/L	3.91	1.20–12.71	0.023			
T1 stage	0.06	0.01–0.28	< 0.001			
T2	0.32	0.12–0.82	0.018			
T3	0.51	0.28–0.89	0.020			
T4	Ref	–				
N0 stage	Ref	–				
N1	4.1	1.71–10.06	0.002			
N2	5.06	2.01–12.76	< 0.001			
N3a	5.72	2.47–13.23	< 0.001			
N3b	10.96	4.80–25.03	< 0.001			
TNM I A	n.e	n.e				
TNM I B	0.13	0.04–0.47	0.002			
TNM II A	0.11	0.03–0.33	< 0.001			
TNM II B	0.18	0.08–0.43	< 0.001			
TNM III A	0.39	0.19–0.80	0.010			
TNM III B	0.37	0.17–0.78	0.010			
TNM III C	Ref	–				
TNM IV	1.11	0.14–8.38	0.915			
Tumor location Antrum vs body	1.38	0.72–2.62	0.326			
Antrum vs fundus/cardia	2.15	1.17–3.95	0.013			
Antrum vs others	0.28	0.03–2.06	0.214			
Clinical local vs locally advanced disease	2.36	1.41–3.95	0.001			
R1 resection	4.27	1.68–10.85	0.002			
Lymphatic invasion	5.40	2.31–12.63	< 0.001			
Vascular invasion	2.38	1.41–3.99	0.001			
Number of total nodes dissected	1.44	1.01–2.05	0.043			
Number of metastatic nodes	1.54	1.30–1.81	< 0.001			
Neoadjuvant chemotherapy	2.93	1.54–5.56	0.001			
Adjuvant chemotherapy	6.78	2.91–15.79	< 0.001			
Thromboembolic complications	7.83	1.02–60.18	0.048			

*HR* hazard ratio, *CI* confidence interval, *LNR* lymph node ratio, *n.e*. not estimable

**Table 4:** Variables associated with Overall Survival (OS) according to the Cox proportional hazards regression model

Variables associated	Univariate analysis			Multivariate analysis		
	HR	95% CI	*p* value	HR	95% CI	*p* value
Age	1.49	1.08–2.06	0.015	1.63	1.02–2.62	0.04
T1	0.11	0.03–0.31	< 0.001	0.21	0.06–0.72	0.02
T2	0.39	0.17–0.88	0.024	0.31	0.09–0.95	
T3	0.47	0.27–0.81	0.002	0.42	0.21–0.85	
T4	Ref	–		Ref	–	
N0	Ref	–		Ref	–	0.0002
N1	2.91	1.36–6.25	0.006	4.53	1.84–11.15	
N2	3.16	1.45–6.90	0.004	5.38	2.08–13.86	
N3a	3.26	1.59–6.68	0.001	3.15	1.31–7.56	
N3b	6.94	3.50–13.77	< 0.001	9.16	3.44–24.35	
TNM I A	0.07	0.02–0.22	< 0.001			
TNM I B	0.19	0.07–0–56	0.003			
TNM II A	0.15	0.05–0.39	< 0.001			
TNM II B	0.19	0.08–0.44	< 0.001			
TNM III A	0.56	0.29–1.06	0.074			
TNM III B	0.43	0.21–0.85	0.017			
TNM IV	1.65	0.21–12.37	0.628			
Tumor size	1.23	0.91–1.66	0.174	0.50	0.33–0.75	0.0008
Antrum/pylorus	Ref	–		Ref	–	0.001
Body	1.78	1.01–3.14	0.044	2.37	1.14–4.93	
Fundus/cardia	1.89	1.05–3.39	0.032	3.94	1.96–7.92	
Others (gastric remnant, multicentric disease)	0.96	0.28–2.95	0.870	1.36	0.27–6.69	
Clinical local Vs	Ref	–				
Locally advanced disease	1.94	1.20–3.12	0.006			
R1 resection	5.33	2.61–10.89	< 0.001			
Ming classification infiltrative	Ref	–				
Expansive	0.38	0.18–0.81	0.012			
Mixed	0.27	0.03–2.01	0.206			
Lymphatic invasion	3.19	1.66–6.11	< 0.001			
Vascular invasion	2.51	1.56–4.04	< 0.001			
LNR	2.22	1.77–2.77	< 0.001			
Positive resection margins	3.53	1.74–7.18	< 0.001			
Length of stay	1.08	1.03–1.12	< 0.001			
Post-operative complications	2.36	1.49–3.75	< 0.001	3.09	1.70–5.65	< 0.001
Post-operative bleeding	2.85	1.46–5.58	0.002			
Anastomotic leakage	2.72	1.09–6.78	0.031			
Neoadjuvant chemotherapy	2.01	1.05–3.84	0.033			
Adjuvant chemotherapy	1.18	0.73–1.94	0.488			

*HR* hazard ratio, *CI* confidence interval, *LNR* lymph node ratio

Age, tumor location, clinical locally advanced stage, T stage, N stage, lymphatic and vascular invasion, LNR, margin status (R1), length of stay, post-operative complications, post-operative bleeding, and anastomotic leakage were significantly associated with OS. At multivariable analysis, age (HR 1.63, 95% CI 1.02–2.62, *p* = 0.041), tumor size (HR 0.50, 95% CI 0.33–0.75, *p* < 0.001), T stage (T1 vs T4: HR 0.21, 95% CI 0.06–0.73, *p* = 0.014; T2 vs T4: HR 0.31, 95% CI 0.10–0.96, *p* = 0.042; T3 vs T4: HR 0.43, 95% CI 0.22–0.85, *p* = 0.016), N stage (N1 vs N0: HR 4.53, 95% CI 1.84–11.16, *p* = 0.001; N2 vs N0: HR 5.38, 95% CI 2.09–13.86, *p* < 0.001; N3a vs N0: HR 3.15, 95% CI 1.31–7.56, *p* = 0.010; N3b vs N0: HR 9.16, 95% CI 3.45–24.36, *p* < 0.001), tumor location (body vs antrum/pylorus: HR 2.37, 95% CI 1.14–4.93, *p* = 0.020; fundus/cardia vs antrum/pylorus: HR 3.95, 95% CI 1.97–7.93, *p* < 0.001), and post-operative complications (HR 3.09, 95% CI 1.69–5.64, *p* < 0.001) were independent predictors of OS.

**Fig. 2:**
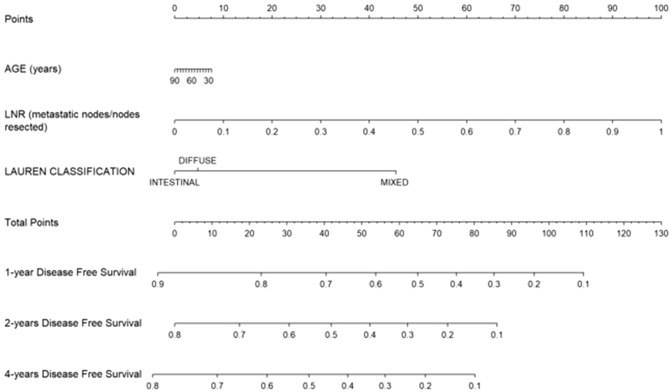
Nomogram predicting disease-free survival

**Fig. 3:**
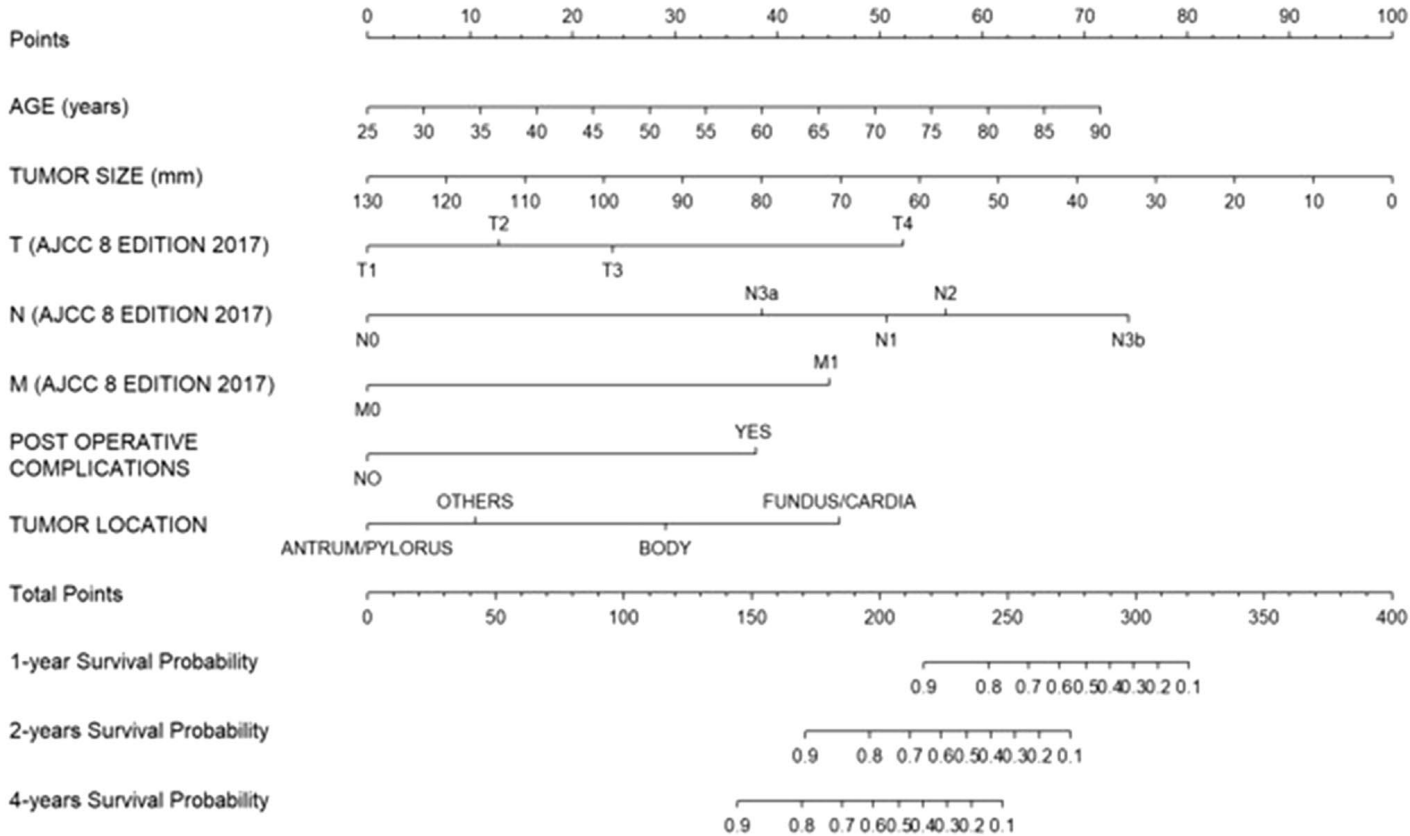
Nomogram predicting overall survival

**Fig. 4:**
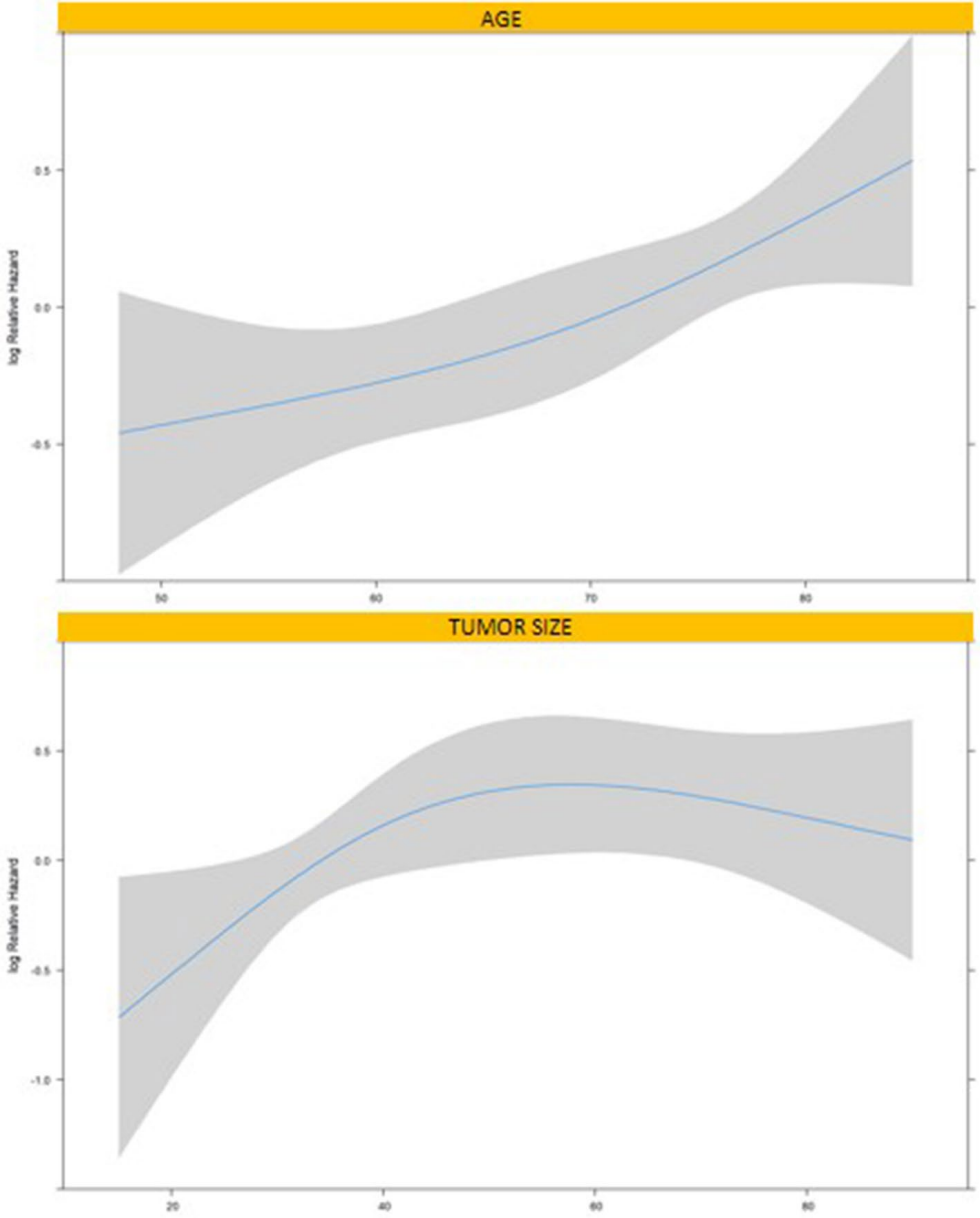
Transformation of continuous variables in univariable analysis using restricted cubic splines relating to age and tumor size

Nomograms to predict DFS (Fig. 2) and OS (Fig. 3) were developed according to variables included in the multivariable Cox regression models. LNR, age and tumor size were used as continuous variables and possible non-linear effects on log HR were explored using restricted cubic splines with 3 knots (Fig. 4). A weighted score was given to each parameter composing the nomograms. The sum of scores was incorporated into an algorithm predicting an individualized OS and DFS. Survival plots for OS and DFS completed the models. For example, a 70-year-old patient with diffuse type GC and LNR = 0.14 would have an estimated DFS of 80% at 12 months of follow-up. This can be deducted by summing the points scored by the patient for every single item included in the nomograms in Fig. 2 and Fig. 3. On the other hand, a 70-year-old patient with a 35 mm tumor located in gastric antrum, T3N2M0, without post-operative morbidity would have a predicted 24 months OS of approximately 70%. On internal validation, both nomograms demonstrated a good discrimination with a Harell’s C-index of 0.77 for OS and 0.71 for DFS.

## Discussion

In addition to the TNM staging system, other factors have been associated with survival after curative-intent surgery for GC. To this point, the TNM system is internationally adopted standard for cancer staging, and GC patients within the same pathological TNM stage often have different survival [7, 11]. In turn, a more accurate prediction of long-term oncological outcomes would be achieved by including other relevant variables, using prediction model such as nomograms. In contrast to TNM staging system, nomograms provide an individual estimation of survival, rather than a stratification in risk groups [14]. Individualized prediction of survival can be useful for several aspects of clinical practice, such as informing patients and families, and recommending a close follow-up or specific treatment in high-risk cases.

In the current study, we developed nomograms based on a single European institution experience of 10 years of treatment of GC. To date, ours is the first Italian nomogram predicting OS for GC in all pathological stages. A previous tool was developed to predict OS for advanced GC in the second line setting, after failure of first line chemotherapy [25]. To our knowledge, this is also the first nomogram that included the effect of post-operative complications on risk of long-term survival and recurrence. The impact of post-operative complications has been documented previously with an observed decreased 5-year OS among individual who experienced post-operative morbidity following curative-intent gastrectomy for GC [12, 26, 27]. Of note, morbidity following surgery for GC is fairly common as Powell et al. reported a post-operative morbidity of 34% within 30 days from surgery [27]. According to these results, in the current study, post-operative morbidity occurred in 41% of patients, and on multivariable analysis, post-operative morbidity was an independent predictor of OS (HR 3.09 1.69–5.64).

Kattan et al. from Memorial Sloan Kettering Cancer Center (MSKCC) first introduced a prognostic model alternative to AJCC/UICC system for GC. The MSKCC nomogram was built on a Western cohort of patients and considered 8 variables associated with disease specific survival (DSS) after curative-intent surgery for GC (age, gender, tumor site and size, Lauren’s classification, depth of tumor invasion, number of metastatic nodes and number of total nodes removed). Although just age, tumor site, depth of invasion and number of metastatic nodes were associated with outcomes, the predictive ability of the MSKCC nomogram was superior to that of the AJCC/UICC system with a C-index of 0.80 versus 0.77 for the TNM (*p* value < 0.001) [14]. However, in external validation in Eastern countries using a Korean cohort of patients, the MSKCC nomogram performed worse than the AJCC/UICC system [16]. To overcome these limitations, Song et al. developed a nomogram with similar variables based on Korean patients, which also demonstrated a better performance to predict OS compared with AJCC/UICC system [16]. To date, several other nomograms have been developed based on Korean, Chinese, Japanese and American datasets. These models share similar variables, with some differences regarding the inclusion of some variables, such as Lauren’s classification, tumor size, LNR and CA 19-9 [5, 11, 15, 17, 18].

Recent literature has repeatedly stressed the importance of proposing simplified prognostic models, which can be easily adapted to different facilities all over the world. Regarding GC, Zheng et al. published a simplified nomogram that was validated into three different patient cohorts (American, Italian and Chinese). This nomogram included common variables from previous nomograms that had been associated with OS (e.g. age, sex, depth of invasion and number of metastatic lymph nodes) [7, 16, 18]. Notably, the nomogram proposed in the current study incorporated almost all variables included in the simplified nomogram proposed by Zheng et al. On multivariable analysis, we noted that age, T stage, number of metastatic nodes as well as tumor size, tumor location and post-operative complications were independent predictors of OS. Unlike other neoplasms in which tumor size is part of AJCC/UICC staging system, GC tumor diameter was not included in TNM system, although several authors have reported its clinical relevance [28–30]. While the specific cut-off for tumor size has varied, several previous reports have confirmed the prognostic role of tumor diameter on overall survival [29–31].

The majority of nomograms have focused on the accurate prediction of OS or DSS. In contrast, only a few nomograms have been developed to predict recurrence risk after gastrectomy for GC. Our data suggest that LNR and Lauren’s classification are associated with recurrence risk after curative-intent surgery for GC. Lai et al. proposed a nomogram to predict DFS for early GC [32]. However, this nomogram had limited usefulness in Western countries where early GC is less common. In 2005, an Italian multicenter study proposed a scoring system incorporating age, N stage, depth of invasion, tumor location and extent of lymphadenectomy to predict recurrence [33]. Subsequently Kim et al. proposed a similar nomogram to predict DFS in which N stage and the extent of lymphadenectomy were replaced by LNR [17]. Recent studies found that LNR was a more accurate predictor of OS rather than N stage [34, 35]. Kim et al. reported that LNR could also be a useful tool to select which patients might benefit of adjuvant therapy after resection [36]. Furthermore, a recent nomogram developed by Ma et al. showed that LNR > 0.335 was associated with early tumor recurrence [37].

In the current study, mixed-type GC had a higher recurrence risk than diffuse al GC (HR 3.01, 95% CI 1.22–7.42). The prognostic role of Lauren’s classification has also been recently reported by Chen et al. who observed a better OS and a lower recurrence risk in patients with intestinal type GC than those with diffuse type GC [38]. Furthermore, Lee et al. reported different patterns of recurrence between intestinal and diffuse/mixed GC. Distant metastases were frequently observed in intestinal tumors, whereas peritoneal recurrence was more common in diffuse/mixed GC [39]. A few studies analysed the role of mixed-type GC, finding more aggressive behaviour and higher risk of nodal metastasis versus diffuse and intestinal type GC [40, 41].

The current study had several limitations. First, the study was based on a retrospective collected data using information from a single Italian institution experience. The cohort of patients was relatively small when compared with similar Asian or American studies, and it covered patients treated in 10 years. GC incidence is decreasing worldwide, especially due to *Helicobacter Pylori* eradication, and in Italy between 2008 and 2016 the incidence decreased up to 2% [2]. Second, all the cohort underwent a traditional open gastric resection and only a minority received perioperative treatment. When performing minimally invasive gastrectomy, our nomograms may be useful since no difference in terms of long-term oncological outcomes was reported when comparing open and a minimally invasive approach [42, 43]. Furthermore, considering the wide diffusion of the multimodal treatment, our study lacks including patients treated with pre- or perioperative treatment. In this setting, the proposed nomograms may be useful mostly in patients treated with upfront resection. Third, the nomograms proposed included mainly post-operative features, and it would not be applicable to the pre-operative setting. Last, the nomograms also require external validation, to evaluate the performance on different cohorts of patients.

## Conclusion

In the current study, we proposed two different nomograms including clinically relevant variables associated with DFS and OS after curative-intent surgery for GC. On internal validation, both nomograms demonstrated a good discrimination. To our knowledge, these are the first nomograms predicting OS and DFS for GC in Italy for all pathological stages. The proposed nomograms may be useful to guide clinician decision-making, as well help identify patients with high recurrence risk or with a poor estimated survival.

## Data Availability

The datasets generated and analyzed during the current study are available from the corresponding author on reasonable request.

## References

[1] Sung H, Ferlay J, Siegel RL, Laversanne M, Soerjomataram I, Jemal A, Bray F (2021). Global cancer statistics 2020: GLOBOCAN estimates of incidence and mortality worldwide for 36 cancers in 185 countries. CA Cancer J Clin.

[2] AIRTUM, AIOM (2020) I numeri del cancro in Italia 2020. https://www.registri-tumori.it/cms/sites/default/files/pubblicazioni/new_NDC2020-operatori-web.pdf. Accessed on 01/10/2020

[3] Spolverato G, Ejaz A, Kim Y, Squires MH, Poultsides GA, Fields RC, Schmidt C, Weber SM, Votanopoulos K, Maithel SK (2014). Rates and patterns of recurrence after curative intent resection for gastric cancer: a United States multi-institutional analysis. J Am Coll Surg.

[4] Deng J, Liang H, Wang D, Sun D, Pan Y, Liu Y (2011). Investigation of the recurrence patterns of gastric cancer following a curative resection. Surg Today.

[5] Liu D, Lu M, Li J, Yang Z, Feng Q, Zhou M, Zhang Z, Shen L (2016). The patterns and timing of recurrence after curative resection for gastric cancer in China. World J Surg Oncol.

[6] Marrelli D, Roviello F, de Manzoni G, Morgagni P, Di Leo A, Saragoni L, De Stefano A, Folli S, Cordiano C, Pinto E (2002). Different patterns of recurrence in gastric cancer depending on Lauren’s histological type: longitudinal study. World J Surg.

[7] Zheng Z-F, Lu J, Wang W, Desiderio J, Li P, Xie J-W, Wang J-B, Lin J-X, Parisi A, Zhou Z-W (2018). Development and external validation of a simplified nomogram predicting individual survival after R0 resection for gastric cancer: an international, multicenter study. Ann Surg Oncol.

[8] Balachandran VP, Gonen M, Smith JJ, DeMatteo RP (2015). Nomograms in oncology: more than meets the eye. Lancet Oncol.

[9] Marchet A, Mocellin S, Ambrosi A, Morgagni P, Garcea D, Marrelli D, Roviello F, de Manzoni G, Minicozzi A, Natalini G (2007). The ratio between metastatic and examined lymph nodes (N ratio) is an independent prognostic factor in gastric cancer regardless of the type of lymphadenectomy: results from an Italian multicentric study in 1853 patients. Ann Surg.

[10] Sun Z, Xu Y, Li DM, Wang ZN, Zhu GL, Huang BJ, Li K, Xu HM (2010). Log odds of positive lymph nodes: a novel prognostic indicator superior to the number-based and the ratio-based N category for gastric cancer patients with R0 resection. Cancer.

[11] Eom BW, Ryu KW, Nam B-H, Park Y, Lee H-J, Kim MC, Cho GS, Kim CY, Ryu SW, Shin DW (2015). Survival nomogram for curatively resected Korean gastric cancer patients: multicenter retrospective analysis with external validation. PLoS ONE.

[12] Jin LX, Sanford DE, Squires MH, Moses LE, Yan Y, Poultsides GA, Votanopoulos KI, Weber SM, Bloomston M, Pawlik TM (2016). Interaction of postoperative morbidity and receipt of adjuvant therapy on long-term survival after resection for gastric adenocarcinoma: results from the US Gastric Cancer Collaborative. Ann Surg Oncol.

[13] Ejaz A, Pawlik TM (2019) Staging systems for gastric cancer: more complex than TNM. Transl Gastroenterol Hepatol 4:44. 10.21037/tgh.2019.05.11 10.21037/tgh.2019.05.11 PMC6624354 31304421

[14] Kattan MW, Karpeh MS, Mazumdar M, Brennan MF (2003). Postoperative nomogram for disease-specific survival after an R0 resection for gastric carcinoma. J Clin Oncol.

[15] Mu G-C, Huang Y, Liu Z-M, Wu X-H, Qin X-G, Chen Z-B (2019). Application value of nomogram and prognostic factors of gastric cancer patients who underwent D2 radical lymphadenectomy. BMC Gastroenterol.

[16] Song KY, Park YG, Jeon HM, Park CH (2014). A nomogram for predicting individual survival of patients with gastric cancer who underwent radical surgery with extended lymph node dissection. Gastric Cancer.

[17] Kim Y, Spolverato G, Ejaz A, Squires MH, Poultsides G, Fields RC, Bloomston M, Weber SM, Votanopoulos K, Acher AW (2015). A nomogram to predict overall survival and disease-free survival after curative resection of gastric adenocarcinoma. Ann Surg Oncol.

[18] Hirabayashi S, Kosugi S, Isobe Y, Nashimoto A, Oda I, Hayashi K, Miyashiro I, Tsujitani S, Kodera Y, Seto Y (2014). Development and external validation of a nomogram for overall survival after curative resection in serosa-negative, locally advanced gastric cancer. Ann Oncol.

[19] Dindo D, Demartines N, Clavien P-A (2004). Classification of surgical complications: a new proposal with evaluation in a cohort of 6336 patients and results of a survey. Ann Surg.

[20] Watanabe T, Itabashi M, Shimada Y, Tanaka S, Ito Y, Ajioka Y, Hamaguchi T, Hyodo I, Igarashi M, Ishida H, Ishihara S, Ishiguro M, Kanemitsu Y, Kokudo N, Muro K, Ochiai A, Oguchi M, Ohkura Y, Saito Y, Sakai Y, Ueno H, Yoshino T, Boku N, Fujimori T, Koinuma N, Morita T, Nishimura G, Sakata Y, Takahashi K, Tsuruta O, Yamaguchi T, Yoshida M, Yamaguchi N, Kotake K, Sugihara K (2015). Japanese Society for Cancer of the Colon and Rectum (JSCCR) guidelines 2014 for treatment of colorectal cancer. Int J Clin Oncol.

[21] Amin MB, Edge SB, Greene FL, Byrd DR, Brookland RK, Washington MK, Gershenwald JE, Compton CC, Hess KR, Sullivan DC (2017). AJCC cancer staging manual.

[22] Harrell FE (2015). Regression modeling strategies: with applications to linear models, logistic and ordinal regression, and survival analysis.

[23] R Development Core Team (2017). R: A language and environment for statistical computing.

[24] FEH J (2020) rms: Regression Modeling Strategies. https://CRAN.R-project.org/package=rms. Accessed on 01/06/2020

[25] Pietrantonio F, Barretta F, Fanotto V, Park SH, Morano F, Fucà G, Niger M, Prisciandaro M, Silvestris N, Bergamo F (2018). Estimating survival probabilities of advanced gastric cancer patients in the second-line setting: the gastric life nomogram. Oncology.

[26] Li Q-G, Li P, Tang D, Chen J, Wang D-R (2013). Impact of postoperative complications on long-term survival after radical resection for gastric cancer. World J Gastroenterol.

[27] Powell A, Coxon AH, Patel N, Chan D, Christian A, Lewis W (2018). Prognostic significance of post-operative morbidity severity score after potentially curative D2 gastrectomy for carcinoma. J Gastrointest Surg.

[28] Adachi Y, Oshiro T, Mori M, Maehara Y, Sugimachi K (1997). Tumor size as a simple prognostic indicator for gastric carcinoma. Ann Surg Oncol.

[29] Saito H, Osaki T, Murakami D, Sakamoto T, Kanaji S, Oro S, Tatebe S, Tsujitani S, Ikeguchi M (2006). Macroscopic tumor size as a simple prognostic indicator in patients with gastric cancer. Am J Surg.

[30] Wang X, Wan F, Pan J, Yu GZ, Chen Y, Wang JJ (2008). Tumor size: a non-neglectable independent prognostic factor for gastric cancer. J Surg Oncol.

[31] Kunisaki C, Makino H, Takagawa R, Oshima T, Nagano Y, Kosaka T, Ono HA, Otsuka Y, Akiyama H, Ichikawa Y (2008). Tumor diameter as a prognostic factor in patients with gastric cancer. Ann Surg Oncol.

[32] Lai JF, Kim S, Kim K, Li C, Oh SJ, Hyung WJ, Rha SY, Chung HC, Choi SH, Wang LB (2009). Prediction of recurrence of early gastric cancer after curative resection. Ann Surg Oncol.

[33] Marrelli D, De Stefano A, de Manzoni G, Morgagni P, Di Leo A, Roviello F (2005). Prediction of recurrence after radical surgery for gastric cancer: a scoring system obtained from a prospective multicenter study. Ann Surg.

[34] Spolverato G, Ejaz A, Kim Y, Squires MH, Poultsides G, Fields RC, Bloomston M, Weber SM, Votanopoulos K, Acher AW (2015). Prognostic performance of different lymph node staging systems after curative intent resection for gastric adenocarcinoma. Ann Surg.

[35] Smith DD, Nelson RA, Schwarz RE (2014). A comparison of five competing lymph node staging schemes in a cohort of resectable gastric cancer patients. Ann Surg Oncol.

[36] Kim Y, Squires MH, Poultsides GA, Fields RC, Weber SM, Votanopoulos KI, Kooby DA, Worhunsky DJ, Jin LX, Hawkins WG (2017). Impact of lymph node ratio in selecting patients with resected gastric cancer for adjuvant therapy. Surgery.

[37] Ma M, Xiao H, Li L, Yin X, Zhou H, Quan H, Ouyang Y, Huang G, Li X, Xiao H (2019). Development and validation of a prognostic nomogram for predicting early recurrence after curative resection of stage II/III gastric cancer. World J Surg Oncol.

[38] Chen Y-C, Fang W-L, Wang R-F, Liu C-A, Yang M-H, Lo S-S, Wu C-W, Li AF-Y, Shyr Y-M, Huang K-H (2016). Clinicopathological variation of Lauren classification in gastric cancer. Pathol Oncol Res.

[39] Lee JH, Chang KK, Yoon C, Tang LH, Strong VE, Yoon SS (2018). Lauren histologic type is the most important factor associated with pattern of recurrence following resection of gastric adenocarcinoma. Ann Surg.

[40] Zheng H-c, Li X-h, Hara T, Masuda S, Yang X-h, Guan Y-f, Takano Y (2008). Mixed-type gastric carcinomas exhibit more aggressive features and indicate the histogenesis of carcinomas. Virchows Arch.

[41] Pyo JH, Lee H, Min B-H, Lee JH, Choi MG, Lee JH, Sohn TS, Bae JM, Kim K-M, Yeon S (2017). Early gastric cancer with a mixed-type Lauren classification is more aggressive and exhibits greater lymph node metastasis. J Gastroenterol.

[42] Spolverato G, Kim Y, Ejaz A, Valero V, Squires MH, Poultsides G, Fields RC, Bloomston M, Weber SM, Acher AW, Votanopoulos K, Schmidt C, Cho CS, Maithel SK, Pawlik TM (2014). A multi-institutional analysis of open versus minimally-invasive surgery for gastric adenocarcinoma: results of the US gastric cancer collaborative. J Gastrointest Surg.

[43] Yu J, Huang C, Sun Y, Su X, Cao H, Hu J, Wang K, Suo J, Tao K, He X, Wei H, Ying M, Hu W, Du X, Hu Y, Liu H, Zheng C, Li P, Xie J, Liu F, Li Z, Zhao G, Yang K, Liu C, Li H, Chen P, Ji J, Li G, Chinese Laparoscopic Gastrointestinal Surgery Study G (2019). Effect of laparoscopic vs open distal gastrectomy on 3-year disease-free survival in patients with locally advanced gastric cancer: the CLASS-01 Randomized Clinical Trial. JAMA.

